# Lymph Node Staging in Perihilar Cholangiocarcinoma: The Key to the Big Picture

**DOI:** 10.3390/curroncol30060438

**Published:** 2023-06-17

**Authors:** Nina A. Rogacka, Tamas Benkö, Fuat H. Saner, Eugen Malamutmann, Moritz Kaths, Juergen W. Treckmann, Dieter Paul Hoyer

**Affiliations:** General, Visceral & Transplantation Surgery, University Hospital Essen, 45133 Essen, Germany

**Keywords:** Klatskin, perihilar cholangiocarcinoma, prognosis, lymph node, multivariable analysis

## Abstract

Klatskin tumors have a bad prognosis despite aggressive therapy. The role and extent of lymph node dissection during surgery is a matter of discussion. This retrospective study analyzes our current experience of surgical treatments in the last decade. **Patients and Methods:** A retrospective single-center analysis of patients (*n* = 317) who underwent surgical treatment for Klatskin tumors. Univariable and multivariable logistic regression and Cox proportional analysis were performed. The primary endpoint was to investigate the role of lymph node metastasis for patient survival after complete tumor resection. The secondary endpoint was the prediction of lymph node status and long-term survival from preoperatively available parameters. **Results:** In patients with negative resection margins, a negative lymph node status was the prognosis-determining factor with a 1-, 3-, and 5-year survival rate of 87.7%, 37%, and 26.4% compared with 69.5%, 13.9%, and 9.3% for lymph-node-positive patients, respectively. Multivariable logistic regression for complete resection and negative lymph node status demonstrated only Bismuth type 4 (*p* = 0.01) and tumor grading (*p* = 0.002) as independent predictors. In multivariate Cox regression analysis, independent predictors of survival after surgery were the preoperative bilirubin level (*p* = 0.03), intraoperative transfusion (*p* = 0.002), and tumor grading (G) (*p* = 0.001). **Conclusion:** Lymph node dissection is of utmost importance for adequate staging in patients undergoing surgery for perihilar cholangiocarcinoma. In spite of extensive surgery, long-term survival is clearly associated with the aggressiveness of the disease.

## 1. Introduction

Perihilar cholangiocarcinomas (PHCCs) are the dominant type of biliary cancer and have an incidence of 1–2 cases per 100,000 persons in the Western world. However, many recent investigations have been carried out to understand this relatively rare malignant disease and identify factors associated with its prognosis. Unfortunately, the prognosis of the disease remains severely limited, and 5-year survival rates between 13.5 and 42% have been reported even for surgically treated cases [1,2,3,4].

However, less than half of all patients can be resected due to locally advanced disease infiltrating adjacent vessels or organs or metastatic disease at the time of diagnosis. Locoregional lymphatic metastases are frequently observed, and a positive lymph node (LN) status has been shown as a dominant prognosis-driving factor in several studies [5]. Unfortunately, to date, it remains preoperatively uncertain which patients have lymph node metastasis.

Treatment recommendations from the current guidelines include surgical extrahepatic bile duct resection combined with partial hepatectomy and systematic locoregional lymphadenectomy at the level of the hepatoduodenal ligament [6], aiming for complete tumor resection. So far, neoadjuvant treatment regimens are not recommended by current guidelines [6]. However, neoadjuvant therapy might be considered in selected cases, e.g., borderline resectable tumors.

In this context, our study aimed to investigate the role of lymph node metastases for patient survival after complete tumor resection as primary endpoint. Moreover, as secondary endpoints, prediction of lymph node status and long-term survival from preoperatively available parameters was investigated.

## 2. Methods

### 2.1. Study Design

We performed a retrospective analysis on patients who underwent surgical treatment for the diagnosis of Klatskin tumor between January 2009 and December 2018 at the University Hospital of Essen. This retrospective study was approved by the local ethics committee (19-8681-BO) and followed the Declaration of Helsinki. Due to the retrospective study design, informed consent was waived.

Data of all patients diagnosed with a Klatskin tumor were extracted from the digital hospital information system. Two independent investigators verified every patient’s diagnosis. Only patients treated surgically were included in this study. Moreover, to create a homogenous cohort of patients, only patients undergoing resection of the bile ducts and liver parenchyma were included. Patients undergoing partial pancreatoduodenectomy were excluded from this study.

### 2.2. Data

The following data were extracted for each patient from the digital hospital information system: Preoperative clinical data: age, gender, weight, height, BMI, ASA Classification (American Society of Anesthesiologist Classification), preoperative biliary stenting, preoperative cholangitis, preoperative laboratory values (bilirubin, INR, creatinine, thrombocytes, gGT, CA19-9), preoperative MELD score, concomitant preoperative medication. Surgery: kind of resection, operative time, transfusion, reconstruction of the portal vein, reconstruction of the hepatic artery. Postoperative pathology: TNM classification (including lymph node count, lymph node status, perineural invasion), current UICC classification (8th edition), tumor size (in cm), histological subtype. Postoperative clinical data: duration of hospital stay, in-house mortality, laboratory values on postoperative day 5 (bilirubin, INR), postoperative complications (hepatic failure, biliary leakage, abscess, bleeding, relaparotomy, kidney failure), survival. We did not include adjuvant treatments (radiotherapy, chemotherapy) in the data because of incompleteness due to several different oncological treatment centers. Moreover, the period under investigation lies ahead of the publication of the BILCAP study, when adjuvant treatments were not routinely established after the resection of biliary tract cancers [7]. By contacting the primary care physicians, we were able to determine the current survival status of the patients.

### 2.3. Definitions

#### 2.3.1. Preoperative Stenting

All ERCPs were reviewed. In the case of insertion of stents into the biliary ducts, which lasted until the surgical procedure, the patients were defined as stented. We performed preoperative biliary drainage (PBD) on most jaundice patients until their total serum bilirubin (TB) level reached 2–5 mg/dL or below. However, in a few patients (~8%), endoscopic therapy was insufficient to achieve this goal. The reasons were technical limitations and, for example, only unilateral stenting.

#### 2.3.2. Preoperative Cholangitis

Preoperative cholangitis was defined as a clinically apparent episode of fever and elevated laboratory infection parameters correlated with laboratory alterations of bilirubin, gGT, or AP, treated with antibiotics or stent replacement. Diagnosis of Cholangitis followed the Tokyo Guidelines [8].

#### 2.3.3. MELD Score

The MELD score (Model for End-stage Liver Disease) indicates the severity of liver disease. The MELD score is based on three laboratory parameters, the most reliable predictors of the progression of severe liver disease: bilirubin, creatinine, and blood clotting time, the latter measured with INR (international normalized ratio). The MELD score is calculated from these parameters as follows: 10 × (0.957 × ln(serum creatinine [mg/dL]) + 0.378 × ln(bilirubin total [mg/dL]) + 1.12 × ln(INR) + 0.643). If dialysis was performed within the last week, the creatinine value is set to 4.0 mg/dL.

#### 2.3.4. Postoperative Liver Failure and Complications

The International Study Group of Liver Surgery (ISGLS) defined PHLF as a postoperatively acquired deterioration in the ability of the liver to maintain its synthetic, excretory, and detoxifying functions, which are characterized by an increased international normalized ratio and concomitant hyperbilirubinemia on or after postoperative day (POD) 5 [9]. Postoperative complications (need to relaparotomy, bleeding complications, bile leakage) were those that appeared within the first postoperative hospital stay.

#### 2.3.5. In-House Mortality

In-House mortality was defined as patient death during the direct postoperative hospital stay, independent of the length of stay. We aimed to include and focus on patients with a complicated postoperative course. Patients dying shortly after discharge or during a second hospital stay for any reason were not included in this definition.

### 2.4. Surgery

Surgical treatment consisted of resection of the extrahepatic bile duct as much as possible from the proximal region to the upper border of the pancreas. Pathologists examined the upper and lower resection margins using frozen specimens. If malignancy was traceable in the resection margins, these were extended. Liver resections were usually carried out in perihilar carcinomas classified as Klatskin type 3 or higher and were carried out as (extended) hemihepatectomy. The goal of surgery was to achieve carcinoma-free resection margins, without any predefined safety margin. All lymphatic and soft tissues were resected within the hepatoduodenal ligament (retroportal nodes and pericholedochal nodes). In cases of macroscopically conspicuous lymph nodes, such were resected up to the celiac trunc nodes and/or posteriosuperior pancreaticoduodenal nodes. Interaortocaval nodes or superioric mesenteric nodes were rated as distant metastases.

The approach to the hilar region consisted of an early division of the distal bile duct for better access to the vascular structures. Ligation and division of the ipsilateral hepatic artery and portal vein were conducted extrahepatically, with sharp division of the bile duct during parenchymal transection. In cases of portal vein invasion in advanced disease, the extirpation of the tumor was performed with an en bloc portal vein resection. In case of contralateral arterial involvement, the case was considered as unresectable disease. An intrahepatic positive bile duct margin was handled by the operating surgeon on a case-by-case basis depending of the intraoperative situs. Published data suggest that in case of a positive margin the extension of resection does not improve survival, and that it is more likely a marker of disease biology [10].

### 2.5. Statistics

Data were analyzed using SPSS 27.0 software (IBM Inc., Armonk, NY, USA). A two-sided *T*-test was performed comparing mean values and the 95% confidence interval (CI) given. Metric variables were compared with Student’s *t*-test or Mann–Whitney U-test. For categorical variables, the groups were compared with a chi-squared test.

Binary logistic regression analysis was used to determine risk factors and other dependencies.

Cox proportional hazard analysis was performed to identify predictors of patient survival.

Factors presenting with a *p*-value < 0.2 in univariable models were used in the multivariable models. The final multivariable models were built with a backward stepwise approach, with *p* < 0.05 as a prerequisite to be included in the model.

A *p*-value of <0.05 was considered statistically significant.

## 3. Results

### 3.1. Study Population

During the study period, 317 patients were treated for Klatskin carcinoma at our department. Of these, 11 patients were not treated with surgery, 111 patients were found to be irresectable upon exploration, 37 patients were treated with (concomitant) pancreatoduodenectomy, and 52 patients had resection without lymphadenectomy or insufficient documentary. These patients were excluded from the trial, so 106 patients remained for analysis.

### 3.2. Demographics

The mean age at surgery was 67.9 ± 8.9 years. The youngest patient in this study was 43 years old, while the oldest patient undergoing surgery for Klatskin tumor was 84 years of age. Most candidates were male (79 (73.8%)). Mean body height was 174 ± 8.3 cm (range 150–193 cm), and mean body weight was 79.9 ± 14 kg, with the resulting BMI being 26.41 ± 3.6 kg/m². The BMI ranged from 15 to 36 kg/m².

The duration from diagnosis until surgical treatment was a median of 23 (7–324) days (one patient was judged irresectable externally without exploration and was send to our clinic nearly one year after diagnosis and treated accordingly). Most patients (73 (68.8%)) had biliary drainage performed before surgical care. Only 15 (14.2%) patients had an episode of clinically apparent cholangitis documented before surgery. A minority of patients (23 (21.7%)) were treated with acetylsalicylic acid as a concomitant medication. All patients were graded using the ASA Classification, with most classified as Stage 2 (31 (29.2%)) or Stage 3 (37 (34.9%)). Preoperative laboratory values are depicted in Table 1.

### 3.3. Surgery

Surgical treatment aimed to create carcinoma-free resection margins. Treatment was performed depending on the Bismuth-Corlette classification [11]. Klatskin type 1 was observed in 15 (14.2%) patients. Type 2 was seen in eight (7.5%) cases. Type 3a and type 3b were observed in 22 (20.8%) patients, respectively, summing up to 44 (41.6%) cases in type 3 tumors. Type 4 was documented in 39 (36.8%) cases.

Therefore, 24 (22.6%) patients were treated via en bloc resection of extrahepatic bile ducts. Additional (left or right) hemihepatectomy was performed in 41 (38.7%) patients, and extended (left or right) hemihepatectomy in 28 (26.4%) patients. Intraoperative blood transfusion was necessary for 10 (9.4%) patients. The portal vein was reconstructed in 13 (12.3%) cases, and an arterial reconstruction was required in 1 (0.9%) patient. In 13 (12.3%) patients, en bloc resection of extrahepatic bile ducts was performed with palliative intention, as irresectability was documented late in the operative procedure after dissection of the bile duct. The median duration for surgery was 284 (126–510) minutes.

### 3.4. Postoperative Tumor Characteristics

All resected specimens were sent to pathology and classified according to TNM and the respective UICC classifications. All UICC stages were reviewed and re-classified for better comparability according to the current classification (eighth edition).

All tumor characteristics are given in Table 2 and Table 3.

### 3.5. Postoperative Complications and Survival

As defined by ISGLS, postoperative liver failure was observed in 38 (35.8%) patients. In total, 21 (19.8%) patients needed relaparotomy to manage complications. Four (3.8%) patients were revised for acute or prolonged bleeding complications. Bile leakage was observed in 16 (15.1%) cases and was handled by surgical revision or drainage. In the postoperative course, 15 (14.2%) patients died during the initial hospital stay (in-house mortality). The overall survival for all resected patients was 73.3%, 33.9%, and 16.8% after 1, 3, and 5 years, respectively.

**Figure 1:**
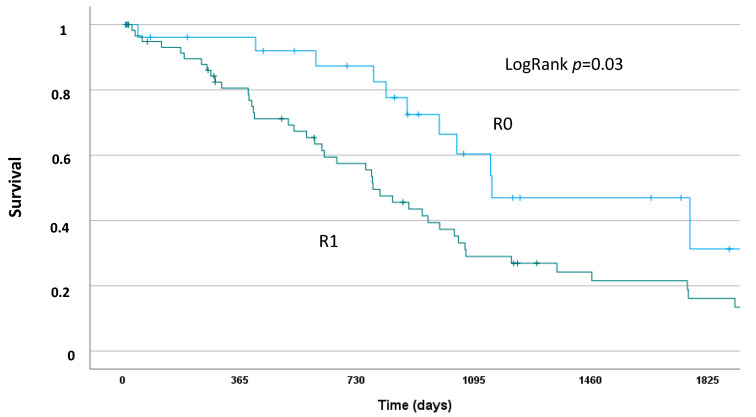
Kaplan–Meier Analysis of postoperative survival for patients with complete resection (R0) and negative lymph node status vs. resected patients with either incomplete resection (R1) and/or positive lymph node status.

**Figure 2:**
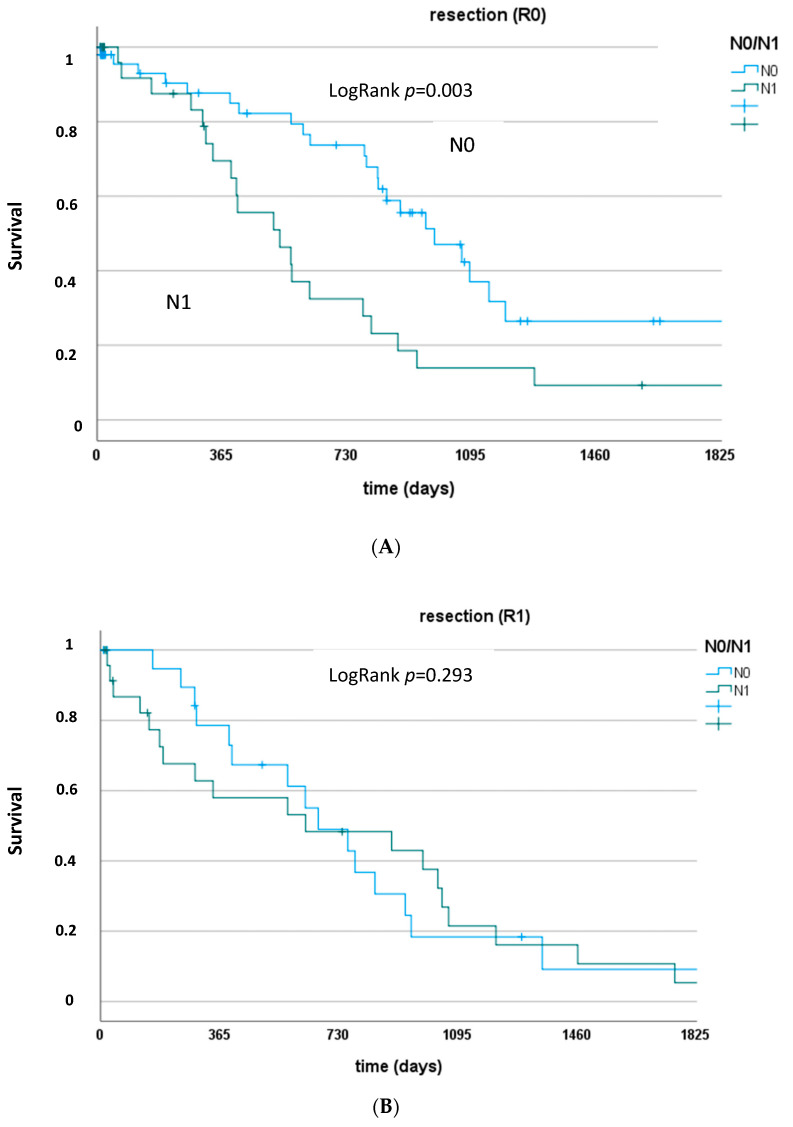
(**A**) Survival rates of patients with complete resection and negative or positive lymph node status (R0 and N0 vs. N1). LogRank *p* = 0.003. (**B**) Survival rates of patients with incomplete resection and negative or positive lymph node status (R1 and N0 vs. N1). LogRank *p* = 0.293.

After censoring for in-house mortality and unsuccessful resections (R2-resection), the 1-, 3-, and 5-year survival rates were 84.5%, 38.7%, and 20%, respectively. Patients with complete resection and negative lymph node status (R0 and N0) had the best postoperative survival with a 1-, 3-, and 5-year survival rate of 96.2%, 60.4%, and 31.3%, respectively (Figure 1 and Figure 2). The median survival of such patients was 1152 (95%CI: 559–1745) days vs. 781 (95%CI: 630–932) days for all other patients with either incomplete resection (R1) or positive lymph node status (N1).

### 3.6. Predictors of Survival

**Figure 3:**
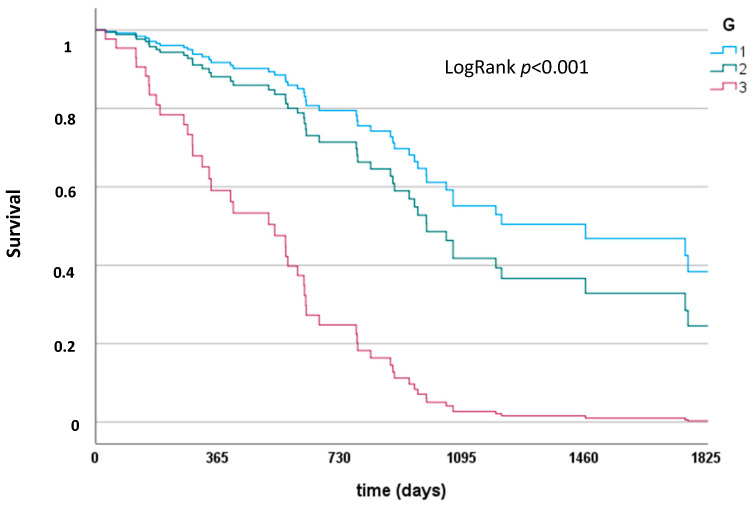
Survival depending on tumor grading (G). LogRank *p* < 0.001.

By uni- and multivariable Cox regression analysis, predictors of postoperative survival (without in-house mortality) were studied (Table 4 and Table 5). Independent predictors of survival after surgery were the preoperative bilirubin level (*p* = 0.03), intraoperative transfusion (*p* = 0.002), and tumor grading (G) (*p* = 0.001). The resection and lymph node status lost their significance in multivariable analysis in favor of the powerful predictive tumor characteristic (G), as shown in Figure 3.

### 3.7. Predictors of Resection Status and Lymph Node Status

We aimed to identify patients eligible for complete resection and negative lymph node status for a thorough risk assessment using preoperatively known patient characteristics. Results of the univariable and multivariable logistic regression analysis are depicted in Table 6 and Table 7. Independent preoperatively known predictors for complete resection and negative lymph nodes were only the clinical Bismuth-Corlette classification (Bismuth type 4 *p* = 0.01) as well as the histopathological grading (*p* = 0.002) of the carcinoma.

## 4. Discussion

This study represents our retrospective experience of surgical treatments for perihilar cholangiocarcinomas in the last decade. The primary endpoint was to investigate the role of lymph node metastasis for patient survival after complete tumor resection.

In cases of lymph node metastases, the outcome was severely compromised and comparable to patients with an incomplete resection (R1). Usually, successful surgical treatment in carcinoma patients is defined as carcinoma-free resection margins. However, in our cohort of patients, the resection margin alone poorly differentiated the long-term survival and was significantly influenced by the lymph node status of the patients. This is why we defined a successful surgical treatment as free resection margin in patients with negative lymph node status. Such patients show the true benefit from surgical treatment and have a clinically relevant long-term prognosis, as documented in Figure 1. This was demonstrated in other patient cohorts as well: Benzing et al. showed that the median survival is not different for patients with lymph node metastases in cases of complete (R0) or incomplete (R1) resection [12]. This underlines the importance of a systematic locoregional lymphadenectomy in patients with perihilar cholangiocarcinoma for a correct staging and individual risk assessment. This fosters the conclusion of the BILCAP Study to apply adjuvant chemotherapy following surgery for biliary tract cancer, at least in all patients with positive lymph nodes [7]. However, aggressive lymphadenectomy does not go without a price to pay:

The example of pancreatic cancer seems appropriate to judge the meaning of an extended lymphadenectomy in oncologic surgery of the upper GI. While a regional lymphadenectomy improves the staging of the disease, resection of secondary or third level lymph nodes would only improve survival without other microscopic disease. Unfortunately, only a minority of patients presumably develop microscopic disease in second or third level lymph nodes without other systemic occurrences. As such, only a small minority of patients will recur with isolated unresected second level metastatic lymph nodes. It has been concluded that removing negative lymph nodes in secondary level lymph nodes will not improve disease control. On the other hand, a higher rate of intra- and postoperative complications have been observed after extended lymphadenectomy in different oncologic entities [13,14,15]. Based on such extrapolation from other entities, it seems appropriate to perform a systematic and careful lymphadenectomy in the primary nodes for proper staging and perform adjuvant therapies in case of any suspicion of microscopic systemic disease. A recent multi-institutional study suggested that the optimal minimal number of resected lymph nodes in perihilar cholangiocarcinoma is four nodes [16], which further highlights a mandatory lymphadenectomy of the primary nodes, without excessive extension of surgery resulting in more complications. Other studies reported a significant influence of the lymph node ratio after resection of perihilar cholangiocarcinoma [17,18], which was not observed in our cohort of patients (data not shown).

From our perspective, the presented data are in accordance with the literature, suggesting a standardized lymphadenectomy of the primary nodes resulting in four lymph nodes in every patient undergoing surgery for perihilar cholangiocarcinoma.

Due to this ongoing discussion, we aimed as a secondary endpoint for the prediction of the lymph node status from preoperatively available parameters, which is of utmost interest from a clinical point of view. Our analysis demonstrated that such predictions are hardly possible: The only independent parameters predictive for this endpoint were the Bismuth-Corlette stage and the histopathological grading. While both were assessable in several preoperative cases, both could not be influenced and represent advanced disease.

On the other hand, this underlines the relevance of precise preoperative diagnostics using cholangiography and concomitant biopsy and should be the substantial basis to inform and advise the patient of the correct expectation of what is achievable by surgery.

Future work might establish neoadjuvant treatment concepts for such patients to improve outcomes after surgery. Unfortunately, successful neoadjuvant concepts are missing so far.

It should be of interest that the best diagnostic tool in cases of biliary tract cancer is still under debate: While some authors suggest percutaneous biliary drainage (PTBD) for superior delineation of tumor involvement of the biliary tree, as well as superior anatomic stent placement [19], it carries the disadvantage of external drains. On the other hand, ERCP is associated with higher rates of stent misplacement, resulting in additional procedures and time delays in surgical treatment. Unfortunately, a multicenter, randomized controlled trial from the Netherlands [20] investigating the best diagnostic tool prematurely ended with increased mortality in the PTBD group compared with the ERCP group. However, several limitations of this study warrant cautious interpretation. Overall and preoperative complications were similar in both groups. Moreover, other studies describe higher rates of infections after ERCP compared with PTBD in patients with advanced biliary tract cancer [21].

In this context, it should be pointed out that preoperative biliary drainage is still under debate, and some data demonstrated that drainage does not affect overall mortality in jaundiced patients [22]. Therefore, non-invasive methods, such as MRCP alone, might be considered for diagnostic workups. However, based on the present data with the tumor grading as an independent predictor of lymph node metastasis and the patient’s overall survival after surgery, a biopsy giving some evidence of the tumor’s characteristics might be of interest preoperatively.

Last, our secondary endpoints included predictive factors of overall patient survival. Regarding the overall survival, we found, as expected, that patients with advanced disease, as represented by lymph node metastasis and incomplete resection margins, did worse. This proves that only patients with a limited disease can be transferred into curative strategies. The 1-, 3-, and 5-year survival rates for curatively resected patients in this study were 84.5%, 38.7%, and 20%. With a 1-year survival rate of 87.7%, patients in this study had an above-average survival rate compared with resected patients in other studies. The 3- and 5-year survival, on the other hand, can be classified as average in the present study situation. The following 1-, 3-, and 5-year survival rates were observed in current studies for curatively resected patients: 40–70%/27–56%/10–42% [23,24,25,26,27].

Additionally, the preoperative level of bilirubin was identified as an independent predictor of overall patient survival, as observed by others [28]. We want to point out that most of our patients are treated with preoperative bile duct stenting during ERCP. This suggests that the preoperative level of bilirubin may effectively reflect the severity of the disease and, therefore, of advanced and complex cases, which, despite preoperative stent insertion, have an incomplete reduction of cholestasis or only partially successful endoscopic drainage (e.g., only one-sided drainage). Intraoperative blood transfusion was also an independent predictor of overall survival after censoring for in-house mortality. This should be seen in the context of the low transfusion rate of only 10% of the population under investigation. Of course, the intraoperative transfusion of RBCs might resemble more complex surgical procedures, and therefore advanced disease. However, other explanations, especially in the context of the overall survival, include transfusion-related immunomodulation (TRIM) leading to diminished immune surveillance and the elusion of micrometastases. Interestingly, the promotion of growth in cancer cells via blood transfusions has been shown in animal models [29]. However, the exact mechanism underlying TRIM remains unclear [30]. Several cell lines (natural killer cells, T cells, suppressor T cells, macrophages, and monocytes) have been suggested to be altered in count and function [31]. This result aligns with other investigations, identifying blood transfusions as an independent predictor of survival in hepatobiliary surgery [32].

Limitations of our study include the monocentric retrospective design and the limited number of patients. The retrospective aspect may introduce selection bias and misclassification or information bias. When relying on individual recall of former exposure to risk variables, the recall may be inaccurate and subject to biases. It can be tough to accurately compare the exposed and the non-exposed. Another relevant limitation of the study is the lack of information regarding applied adjuvant therapies. Since reliable data on that matter were only collected within our hospital postoperatively, disease-specific survival could not be collected in all cases and therefore had to be reported as overall survival. Additionally, one-third of the existing cohort had an ASA classification of three at the time of surgery, suggesting relevant comorbidities with an impact on overall survival. On the other hand, it can be assumed that other study cohorts reporting on cohorts of the same average age at diagnosis underly the same bias, so there is still comparability. However, there are also strengths of our study. First, all patients were treated by a few different surgeons and anesthesiologists. All underwent standardized operations and anesthesia procedures, and all patients were followed at a dedicated ICU. Moreover, a cohort of 107 patients seems large enough to conclude relevance to treating a rare disease in a medical context.

Our results demonstrate the necessity and feasibility of surgical treatment in Klatskin carcinoma, with surgery as the only option for a curative treatment concept. However, a remarkably high number of patients were sent for surgery and found unresectable upon surgical exploration. The treatment of Klatskin carcinomas remains challenging due to the aggressiveness of the disease, its late diagnosis, and its complex anatomical location.

In conclusion, our data demonstrate the impact of resection margins in combination with the lymph node status for the survival of patients undergoing surgery for Klatskin tumors. Unfortunately, a good prediction of this subgroup of patients using preoperatively known parameters is limited.

## Figures and Tables

**Table 1:** Preoperative laboratory values.

	Median	Range	Standard Values
Bilirubin (mg/dL)	1.3	0.2–23.9	≤1.1 mg/dL
Creatinine (mg/dL)	0.98	0.47–6.8	(m) ≤ 1.1 mg/dL (f) ≤ 0.9 mg/dL
INR	1	0.83–3.33	0.7–1.2
gGT (U/L)	393	13–3094	(m) ≤ 55 U/L (f) ≤ 38 U/L
CA19-9 (U/L)	103.9	1–422,490	≤37 U/L
Platelets (/nL)	296	14–966	150–400/nL

**Table 2:** Tumor characteristics (TNM classification) of all resected patients.

	*n*	%
T1	15	14.2
T2	73	68.9
T3	13	12.3
T4	3	2.8
G1	4	3.8
G2	70	66
G3	26	24.5
N-	60	56.6
N+	46	43.4
L0	79	74.5
L1	22	20.8
V0	92	86.8
V1	11	10.4
Pn0	18	16.9
Pn1	61	57.4
R0	52	49.1
R1	49	46.2
R2	5	4.7

T = tumor size, G= tumor grade, N = lymph node, L = lymphatic invasion, V = vein invasion, Pn = perineural invasion, R = resection margin. Missing data: T (2 missing), G (6 missing), L (5 missing), V (3 missing), Pn (27 missing).

**Table 3:** UICC Classification (8th Edition).

UICC class	Number (%)
1	11 (10.4%)
2	40 (37.7%)
3	54 (50.9%)
4	1 (0.9%)

**Table 4:** Results of univariable Cox regression analysis for patient survival (without IHM).

	*p*-Value	Hazard Ratio	Lower 95% CI	Upper 95% CI
Duration from diagnosis to surgery	0.19	1.004	0.999	1.009
Age	0.66	0.993	0.96	1.03
Gender	0.29	1.39	0.74	2.6
Weight	0.24	0.98	0.97	1.01
Height	0.72	0.99	0.96	1.03
BMI	0.22	1.96	0.89	1.03
ASA Classification	0.058	1.97	0.98	3.98
Preoperative Stenting	0.27	1.51	0.73	3.11
Cholangitis before surgery	0.83	0.92	0.44	1.94
ASS medication	0.29	1.41	0.74	2.68
CA 19-9 preoperatively	0.4	1	1	1
Bilirubin preoperatively	0.11	1.09	0.98	1.23
Creatinine preoperatively	0.89	0.91	0.26	3.25
INR preoperatively	0.39	2.01	0.41	9.96
Platelets preoperatively	0.32	1.001	0.999	1.004
gGT preoperatively	0.131	1	0.99	1
MELD score preoperatively	0.11	1.03	0.99	1.08
Duration of surgery	0.713	1.001	0.997	1.004
**Transfusion**	**0.003**	**4.21**	**1.62**	**10.93**
Portal reconstruction	0.21	1.73	0.74	4.09
T	0.22	1.29	0.86	1.96
**G**	**0.008**	**2.04**	**1.21**	**3.44**
Lymph node status positive	0.13	1.49	0.89	2.49
Lymph node count	0.63	0.98	0.88	1.08
L	0.18	1.56	0.81	2.99
V	0.25	1.56	0.74	3.31
**Pn**	**0.007**	**2.98**	**1.35**	**6.59**
R0 (negative resection margin)	0.72	1.09	0.69	1.704
**R0 and negative lymph node status**	**0.032**	**0.49**	**0.26**	**0.94**
**Liver failure (ISGLS)**	**0.04**	**1.74**	**1.02**	**2.96**
Bismuth-Corlette Type	0.28	1.16	0.89	1.52
Re-laparotomy for complications	0.85	1.06	0.55	2.05
Bile leak	0.2	1.68	0.75	3.76
Postoperative fluid collection/abscess	0.353	1.49	0.64	3.51
Postoperative bleeding	0.19	0.26	0.04	1.9
Postoperative kidney failure	0.38	1.89	0.46	7.8

Statistically significant parameters in bold.

**Table 5:** Results of multivariable Cox regression analysis for patient survival (without IHM).

	*p*-Value	Hazard Ratio	Lower 95% CI	Upper 95% CI
**Bilirubin preoperatively**	**0.03**	**1.21**	**1.02**	**1.42**
**Intraoperative blood transfusions**	**0.002**	**5.1**	**1.83**	**14.01**
**G**	**0.001**	**3.1**	**1.57**	**6.06**

Statistically significant parameters in bold.

**Table 6:** Results of univariable logistic regression analysis for complete resection and negative lymph node status.

	*p*-Value	Odds Ratio	Lower 95% CI	Upper 95% CI
Duration from diagnosis to surgery	0.24	1.01	0.99	1.03
Age	0.15	0.97	0.92	1.01
Gender	0.25	1.72	0.69	4.28
Weight	0.91	1.002	0.97	1.03
Height	0.53	0.98	0.94	1.04
BMI	0.49	1.04	0.93	1.17
ASA Classification	0.67	0.82	0.33	2.02
Preoperative Stenting	0.85	0.88	0.25	3.09
Cholangitis before surgery	0.89	0.92	0.29	2.94
ASS medication	0.3	0.59	0.23	1.53
CA 19-9 preoperatively	0.6	1.001	0.99	1.003
Bilirubin preoperatively	0.74	1.04	0.83	1.29
Creatinine preoperatively	0.77	1.34	0.19	9.39
INR preoperatively	0.69	1.91	0.08	45.79
Platelets preoperatively	0.79	1	0.99	1.004
gGT preoperatively	0.87	1	0.99	1.001
MELD score preoperatively	0.65	1.02	0.95	1.09
Duration of surgery	0.22	0.99	0.99	1.002
Transfusion	0.15	4.65	0.56	38.29
Portal reconstruction	0.94	1.05	0.29	3.7
T	0.43	1.33	0.65	2.72
**G**	**0.003**	**0.16**	**0.05**	**0.53**
Lymph node count	0.57	1.05	0.88	1.26
L	0.12	0.39	0.12	1.27
V	0.15	0.22	0.03	1.78
**Pn**	**0.01**	**0.24**	**0.08**	**0.74**
Tumor size in cm	0.35	1.11	0.89	1.36
Bismuth-Corlette classification	0.09			
Bismuth type 1	0.11	0.34	0.09	1.27
Bismuth type 2	0.69	1.58	0.17	14.99
Bismuth type 3	0.1	0.37	0.11	1.22
**Bismuth type 4**	**0.01**	**0.23**	**0.07**	**0.73**

Statistically significant parameters in bold.

**Table 7:** Results of multivariable logistic regression analysis for complete resection and negative lymph node status.

	*p*-Value	Odds Ratio	Lower 95% CI	Upper 95% CI
Bismuth-Corlette classification	0.11			
Bismuth type 1	0.19	0.38	0.09	1.59
Bismuth type 2	0.83	1.29	0.13	13.08
Bismuth type 3	0.21	0.43	0.11	1.63
**Bismuth type 4**	**0.01**	**0.18**	**0.05**	**0.67**
**G**	**0.002**	**0.14**	**0.04**	**0.49**

Statistically significant parameters in bold.

## Data Availability

The data presented in this study is available on request from the corresponding author.

## Abbreviations

ASA Classification	American Society of Anesthesiologists Classification
BILCAP study	Capecitabine compared with observation in resected biliary tract cancer
BMI	Body-Mass-Index
CA 19-9	Carbohydrate Antigen 19-9
CI	Confidence intervals (95%)
CT	Computed tomography
EK	Erythrocyte concentrate
G	Tumor grade
gGT	Gamma-glutamyl transferase
ICU	Intermediate care unit
INR	International Normalized Ratio
ISGLS	International Study Group of Liver Surgery
LN	Lymph node
MELD	Model for End-Stage Liver Disease
N	Node
OR	Odds Ratio
PBD	Preoperative biliary drainage
PHCC	Perihilar cholangiocarcinoma
PHLF	Posthepatectomy liver failure
PBTC	Percutaneous biliary transhepatic cholangiography
POD 5	5 postoperative days
Pn	Perineural invasion
R	Resection margin
T	Tumor size
UICC/AJCC	Union for International Center Control/American Joint Committee on Cancer
V	Vein invasion
